# The clue is in the lipid A: Rapid detection of colistin resistance

**DOI:** 10.1371/journal.ppat.1008331

**Published:** 2020-04-09

**Authors:** R. Christopher D. Furniss, Markus Kostrzewa, Despoina A. I. Mavridou, Gerald Larrouy-Maumus

**Affiliations:** 1 MRC Centre for Molecular Bacteriology and Infection, Department of Life Sciences, Imperial College London, London, United Kingdom; 2 Bruker Daltonik GmbH, Bremen, Germany; 3 Department of Molecular Biosciences, University of Texas at Austin, Austin, Texas, United States of America; Tufts University School of Medicine, UNITED STATES

## Matrix-assisted laser desorption/ionization-time of flight mass spectrometry is an emerging technology for the detection of antimicrobial resistance

Antimicrobial resistance (AMR) is one of the biggest public health concerns of our time [1]. Currently, resistance has been reported for almost all available antibiotics, from first-line to last-resort drugs [2]. AMR restricts treatment options, leads to increased morbidity and mortality of hospitalized patients and imposes a substantial financial burden on healthcare systems worldwide [3]. The limited pipeline of new antimicrobials and the continuous emergence of multidrug-resistant (MDR) organisms needs to be countered by novel antibacterial strategies coupled with rapid diagnostics to detect resistance, particularly in the case of broad-acting agents such as β-lactams and polymyxins.

Traditionally, antimicrobial susceptibility testing is performed using culture-based methods, like minimum inhibitory concentration (MIC) determination assays, a lengthy process that is prone to misleading results, for example, in the case of clinical isolates that produce large amounts of capsule or biofilm. By contrast, polymerase-chain-reaction (PCR)-based tests like multiplex PCR allow the rapid identification of specific resistance factors but cannot predict susceptibility and are entirely insensitive to uncharacterized mechanisms of resistance. At the same time, despite the extensive progress of genotypic sequence-based approaches, such as long-read nanopore sequencing, these cannot yet be used as first-line diagnostics in part because genotype-to-phenotype predictions remain challenging [4,5].

With matrix-assisted laser desorption/ionization-time of flight (MALDI-TOF) mass spectrometry being used routinely for bacterial identification in clinical settings, a range of MALDI-TOF-based diagnostics for the rapid detection of AMR are starting to be implemented [6,7]. More specifically, it is possible to discriminate some lineages of methicillin-resistant *Staphylococcus aureus*, identify certain strains of enterobacteria carrying plasmid-encoded β-lactamases from the *Klebsiella pneumoniae* carbapenemase (KPC) family of enzymes, and detect *Bacteroides fragilis* strains that are resistant to β-lactams at the same time as performing bacterial identification. Alongside these identification-based approaches, efforts to identify both aminoglycoside and quinolone resistance using MALDI-TOF mass spectrometry have also been made. As resistance to these agents can arise through enzymatic modification of the antibiotic, this modification can be directly detected following incubation of the drug of interest with resistant bacteria [8,9]. Nonetheless, probably the most successful application of MALDI-TOF mass spectrometry for AMR detection to date has been functional screening for β-lactamase activity. Bacteria are mixed with a β-lactam compound, and an increase in the expected mass of the antibiotic by a mass-to-charge ratio (*m/z*) of 18, corresponding to the mass of a water molecule, indicates hydrolysis of the β-lactam ring by an active β-lactamase. This method is approaching use in clinical settings through the development of the MBT STAR-Carba IVD kit for the detection of carbapenemases, led by Bruker. Finally, MALDI-TOF-based identification of resistance has the potential to move beyond specific mechanisms and antibiotics through universal methods based on the indirect detection of bacterial growth by mass spectrometry. These approaches are currently in preliminary stages but have been attempted through the development of the MBT-ASTRA and DOT-MGA assays [10–12].

## MALDI-TOF mass spectrometry can be used to characterize the lipid A of bacteria

Polymyxin antibiotics (e.g., polymyxin B, colistin) are extremely valuable drugs of last resort that are used to treat challenging bacterial infections [13]. As MDR bacterial strains become more prevalent, the use of colistin is an increasingly important way of managing infections caused by these organisms [14]. This increase in the use of colistin, together with the presence of mobile colistin resistance (MCR) enzymes, which were discovered in 2016 [15], has contributed to the spread of colistin resistance in gram-negative bacteria and the emergence of extensively drug-resistant organisms [16]. Therefore, as with β-lactamase-mediated resistance, fast detection of colistin resistance is crucial to improved patient outcomes.

**Fig 1:**
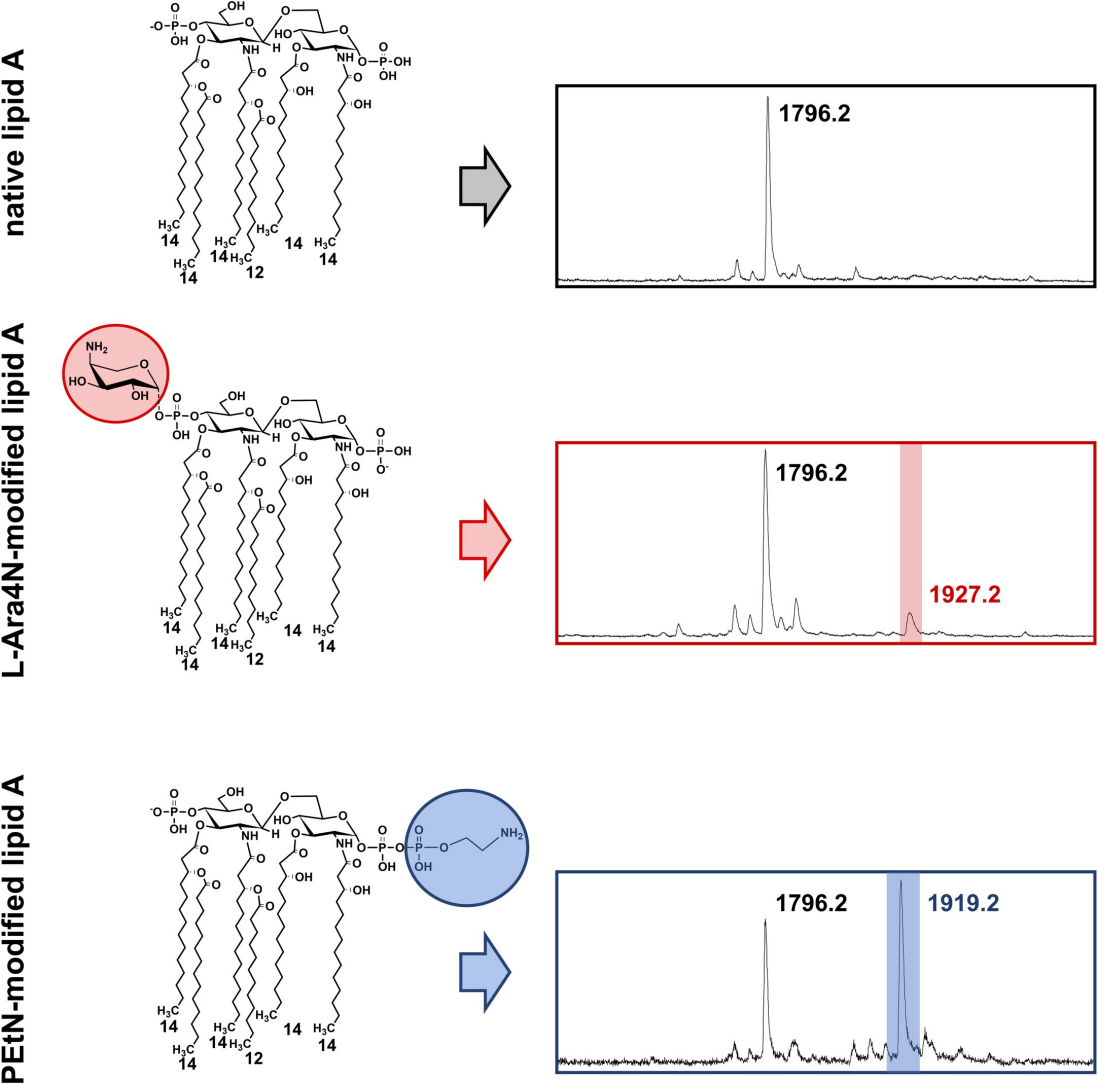
**(Left column) Structures of the native (top) and modified (middle and bottom) lipid A portion of the *Escherichia coli* LPS. L-Ara4N (marked in red) and PEtN (marked in blue) modifications to lipid A cause resistance to polymyxin antibiotics.** L-Ara4N modification of the 4ˊ-phosphate of native lipid A is characteristic of colistin-resistant *E*. *coli* strains carrying chromosomal mutations; this modification often co-occurs with PEtN modification of the 1-phosphate of the lipid A structure. Lipid A from strains expressing MCR enzymes is only modified by PEtN. **(Right column) Lipid A spectra obtained from intact *E*. *coli* colonies using the MALDI Biotyper Sirius system (Bruker Daltonics) and the adapted MALDIxin protocol [18].** Spectra obtained from colistin-susceptible strains have one major peak at *m/z* 1,796.2 (top), corresponding to native lipid A. Depending on the mechanism of colistin resistance, spectra from colistin-resistant strains have additional peaks at *m/z* 1,927.2 (middle, marked in red), due to L-Ara4N modification of lipid A, and/or at *m/z* 1,919.2 (bottom, marked in blue), due to PEtN modification of lipid A. LPS, lipopolysaccharide; L-Ara4N, 4-amino-4-deoxy-L-arabinose; PEtN, phosphoethanolamine; MCR, mobile colistin resistance; *m/z*, mass-to-charge ratio.

Determination of colistin MIC values by broth microdilution, the current gold standard for polymyxin susceptibility testing, is laborious, time-consuming, and sometimes unreliable [15], while PCR-based approaches cannot keep pace with the emergence of novel *mcr* genes. In most gram-negative bacteria, colistin resistance arises from chemical modifications to the lipid A portion of their lipopolysaccharide (LPS), mediated by chromosomally encoded mutations or the activity of MCR proteins [17]. Specifically, the addition of a 4-amino-4-deoxy-L-arabinose (L-Ara4N) and/or a phosphoethanolamine (PEtN) moiety to the 4ˊ- and/or 1-phosphate of native lipid A, respectively, results in decreased binding of colistin to the LPS [17] (Fig 1, left column) and emergence of resistance.

The demonstration that lipid A from several gram-negative bacterial species can be detected and its structure characterized by MALDI-TOF mass spectrometry using intact bacterial colonies [19] laid the groundwork for many of the subsequent studies tackling the development of MALDI-TOF-based diagnostics for the rapid detection of colistin resistance. Notably, unlike bacterial identification or detection of β-lactamase activity, which require sample analysis using the positive-ion mode, this approach relies on operating the mass spectrometer in the negative-ion mode, which was not possible on the instruments in routine clinical use until recently [18].

### The MALDIxin test detects colistin resistance from intact *Escherichia coli* colonies

Chromosomally encoded and MCR-mediated colistin resistance are both prevalent in *Escherichia coli* clinical isolates [15]. As such, this organism was initially used to demonstrate that rapid detection of resistance is feasible by MALDI-TOF mass spectrometry and to develop the MALDIxin test [20]. The sample preparation step for this approach was shown to be fast and simple. A bacterial colony was suspended and washed in ultra-pure water and subsequently mixed with a super-2,5-dihydroxybenzoic acid (sDHB) matrix directly on the target of a research MALDI-TOF instrument. Data collection using the negative-ion mode allowed direct assessment of whether the tested strain was susceptible or resistant to colistin in less than 15 minutes. For colistin-susceptible strains, only one major peak at *m/z* 1,796.2, corresponding to native lipid A, was detected, whereas for colistin-resistant strains, an additional peak at *m/z* 1,919.2 was present; the latter arises from the addition of a PEtN moiety to the 1-phosphate group of lipid A (Fig 1, left column, bottom panel). A metric termed polymyxin resistance ratio (PRR), i.e., the ratio of the sum of the intensities of peaks associated with modified lipid A over the intensity of the peak of native lipid A, was introduced and validated on a panel of *E*. *coli* strains; colistin-susceptible strains had a PRR of zero, whereas colistin-resistant strains had a positive PRR. Blind validation of the method using a collection of 78 *E*. *coli* isolates further supported that MALDI-TOF-based diagnostics hold great promise for rapid, high-throughput and low-cost detection of colistin resistance.

### MALDI-TOF mass spectrometry detects resistance in other gram-negative bacterial species

As modification of lipid A is the most common mechanism of colistin resistance in gram-negative bacteria [17], and the lipid A structure of several species can be characterized by MALDI-TOF mass spectrometry [19], the MALDIxin approach can be applied to other gram-negative pathogens in which colistin resistance is a great challenge. Table 1 summarizes the expected peaks corresponding to native and modified lipid A for organisms in which colistin resistance is prevalent and this approach has been tested. The MALDIxin test is particularly effective for *Acinetobacter baumannii*, successfully detecting resistant strains with minimal sample preparation [21], as well as *K*. *pneumoniae*, for which, despite the requirement for a brief mild acid hydrolysis step, this method provides rapid and accurate identification of colistin resistance [22]. Although acid hydrolysis of the sample was not initially performed for *E*. *coli* and *A*. *baumannii* [20,21], routine application of this step during sample preparation results in improved signal-to-noise ratio, especially for more complex bacteria like *K*. *pneumoniae* [22] while allowing uniform sample processing of any organism of interest.

**Table 1:** *m/z* values rounded to the nearest decimal for native and modified lipid A peaks obtained by MALDI-TOF mass spectrometry from intact colonies of gram-negative bacterial pathogens that are commonly resistant to colistin.

Organism	Native lipid A peak(s) (*m/z*)	Modified lipid A peak(s) (*m/z*)	Lipid A modification
*Escherichia coli*	1,796	1,927	addition of L-Ara4N to the 4ˊ-phosphate of lipid A
		1,919	addition of PEtN to the 1-phosphate of lipid A
*Klebsiella pneumoniae*	1,824; 1,840 2,062; 2,078	1,971; 2,209	addition of L-Ara4N to the 4ˊ-phosphate of lipid A
		1,963; 2,201	addition of PEtN to the 1-phosphate of lipid A
*Salmonella enterica*	1,796 2,034	1,927; 2,165	addition of L-Ara4N to the 4ˊ-phosphate of lipid A
		1,919; 2,157	addition of PEtN to the 1-phosphate of lipid A
*Acinetobacter baumannii*	1,728 1,910	2,033	addition of PEtN to the 1-phosphate of lipid A

The “Lipid A modification” column provides information on the nature of the chemical modification resulting in the presence of each modified lipid A peak in the recorded mass spectra. For *Escherichia coli*, the L-Ara4N modification of lipid A is only detected if the bacterial sample is subjected to mild acid hydrolysis prior to data collection [18] and hence was not observed in the initial MALDIxin test [20]. Values presented for *Salmonella enterica* were recently recorded in the Larrouy-Maumus laboratory. L-Ara4N, 4-amino-4-deoxy-L-arabinose; MALDI-TOF, matrix-assisted laser desorption/ionization-time of flight *m*/*z*, mass-to-charge ratio; PEtN, phosphoethanolamine.

In addition to the MALDIxin test, an alternative (albeit more time-consuming) strategy for lipid A characterization in the context of MALDI-TOF-based diagnostics was recently presented [23]. This approach relies on extraction of lipid A from bacterial samples followed by MALDI-TOF analysis of the extracts and was shown to allow the pairing of bacterial identification [24] with colistin resistance detection [23]. The method has since been employed in a prospective study monitoring colistin susceptibility in clinical *A*. *baumannii* isolates, proving that these approaches have clinical utility [25,26].

### MALDI-TOF-based diagnostics can be used for rapid detection of colistin resistance in clinical settings

**Fig 2:**
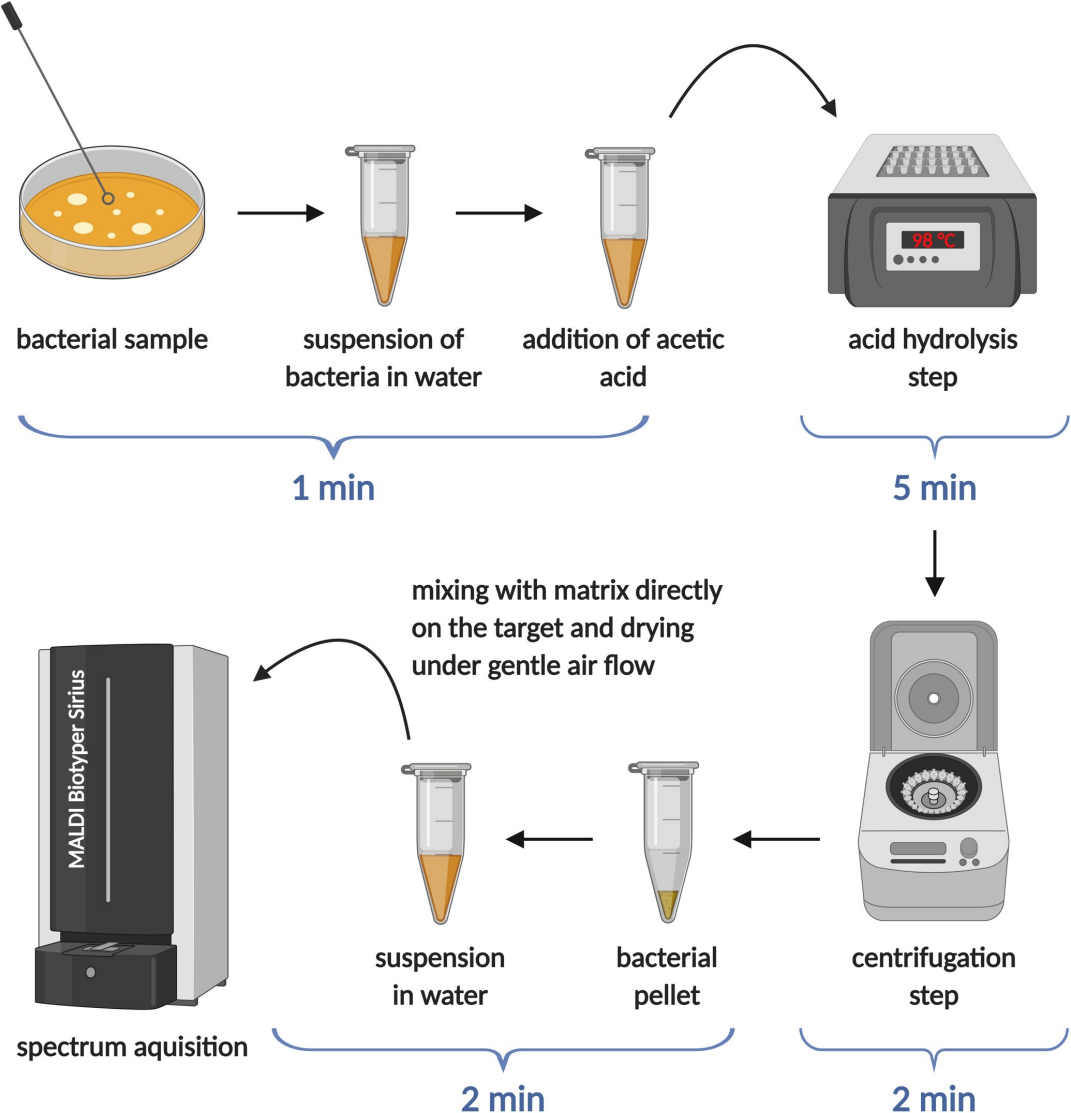
Schematic diagram of the sample preparation process for the adapted MALDIxin test performed on the MALDI Biotyper Sirius system (Bruker Daltonics). The total sample processing time using this approach is less than 15 minutes, allowing for immediate assessment of whether bacteria are resistant to polymyxin antibiotics, independent of the mechanism of resistance. In the published feasibility study [18], bacterial colonies were grown overnight on solid medium; in future clinical applications, bacteria could be obtained directly from biological samples, such as urine or blood. *Figure created with BioRender*.

Although fast and effective, all MALDI-TOF-based methods for detecting colistin resistance were initially developed using research mass spectrometers and were not tested on the instruments commonly used in clinical settings. The main reason for this was that until recently, clinically used mass spectrometers could not operate in the negative-ion mode. The latest model of the widely used MALDI Biotyper system by Bruker Daltonics, the MALDI Biotyper Sirius, is equipped with a negative-ion mode, permitting the recent adaptation of the MALDIxin test for the type of instruments used in hospitals [18].The sample preparation step was optimized for *E*. *coli* strains to maximize the signal-to-noise ratio without compromising the speed of the method (Fig 2). Calculation of PRR values from recorded spectra, taking into account the peaks arising from the addition of PEtN and/or L-Ara4N moieties to lipid A (Fig 1, right column and Table 1), allowed rapid and accurate identification of colistin resistance irrespective of its genetic basis in clinical *E*. *coli* isolates. As before, colistin-susceptible strains gave a PRR value of zero, and positive PRR values indicated resistance to colistin. Ultimately, this feasibility study showed that MALDI-TOF-based detection of colistin resistance using a clinical mass spectrometer can generate an unambiguous yes/no output, thus laying the foundations for a rapid diagnostic test that will be readily accessible to most clinical microbiology laboratories.

### Future directions

There is no doubt that MALDI-TOF-based approaches offer considerable promise for the development of rapid diagnostics for the detection of colistin resistance. The recent advances described here allow the introduction of such methods in clinical settings with the hope that they might eventually become the gold standard for polymyxin susceptibility testing. To achieve this, it is important to expand the range of gram-negative bacteria for which MALDI-TOF-based methods can be applied. This would include tests on organisms that have not been extensively characterized by MALDI-TOF mass spectrometry, like *Pseudomonas*, *Enterobacter*, *Citrobacter*, and *Burkholderia* spp., and further optimization of the sample preparation pipeline so that clinical instruments like the MALDI Biotyper Sirius system would be effectively used for all organisms of interest.

For such approaches to truly reach the clinic, further validation in hospital settings using large numbers of clinical isolates will be essential in order to pinpoint any discrepancies between MIC data and MALDI-TOF-based diagnostics (for example, it is known that in rare cases, colistin resistance in *A*. *baumannii* is due to total absence of lipid A [27] or efflux pump activity [28]). Large-scale testing will also promote the delineation of specific ranges for the metrics used to analyze and evaluate the acquired spectra, enabling clinical microbiologists to make confident calls regarding susceptibility or resistance to colistin in a similar manner as when using the antibiotic breakpoints defined by the European Committee for Antimicrobial Susceptibility Testing (EUCAST). This will have to be achieved in tandem with automation of the data analysis process, which will permit high-throughput processing of the samples. Some effort has already been made towards this in a study showing that machine learning algorithms and MALDI-TOF mass spectrometry can be used to detect colistin-resistant *Acinetobacter* and *Klebsiella* spp. in complex polymicrobial mixtures [29].

Overall, MALDI-TOF-based diagnostics applied for the detection of polymyxin resistance offer major advantages and could become a clinical reality. The strength of these approaches is that they could serve as a first-line strategy to rapidly, reliably, and cost-effectively identify colistin resistance with minimal changes to existing clinical equipment and workflows. It is expected that MALDI-TOF mass spectrometers with negative-ion mode capabilities approved for routine clinical use will make their way into hospital laboratories as part of the regular equipment upgrade cycle, allowing the implementation of this approach alongside existing positive-ion mode applications. If required for epidemiological purposes, in-depth characterization of the isolated strains could be pursued at a second stage using culture-, PCR- or sequencing-based methods. This type of pipeline would ensure that treatment of patients with challenging MDR gram-negative infections happens as efficiently as possible, improving patient outcomes without compromising the necessary epidemiological component of clinical microbiology.
